# Through the Looking Glass: Genome, Phenome, and Interactome of *Salmonella enterica*

**DOI:** 10.3390/pathogens11050581

**Published:** 2022-05-14

**Authors:** Jean Guard

**Affiliations:** U. S. Department of Agriculture, Agricultural Research Service, U. S. National Poultry Research Center, 950 College Station Road, Athens, GA 30605, USA; jean.guard@usda.gov; Tel.: +1-706-202-1541

**Keywords:** *Salmonella enterica*, interactome, phenotype, genotype, serotype, speciation, whole genome sequencing, multi-locus sequence typing, salmonellosis, epidemiology

## Abstract

This review revisits previous concepts on biological phenomenon contributing to the success of the *Salmonella enterica* subspecies I as a pathogen and expands upon them to include progress in epidemiology based on whole genome sequencing (WGS). Discussion goes beyond epidemiological uses of WGS to consider how phenotype, which is the biological character of an organism, can be correlated with its genotype to develop a knowledge of the interactome. Deciphering genome interactions with proteins, the impact of metabolic flux, epigenetic modifications, and other complex biochemical processes will lead to new therapeutics, control measures, environmental remediations, and improved design of vaccines.

## 1. Introduction

The genus *Salmonella* has two distinct species, namely *S. bongori* and *S. enterica*. However, foodborne pathogens are, for the most part, found in only one of six subspecies of *S. enterica*, namely *S. enterica* subspecies I. The other *S. enterica* subspecies are II (salamae), IIIa (arizonae), IIIb (diarizonae), IV (houtenae), and VI (indica). There are some instances where subspecies II–VI cause illness, but overall, they are infrequently encountered as public health issues and are associated with wildlife-centered occupations, reptiles, and animals that are less often used for food [1,2,3]. There is concern that evolution of the genus *Salmonella* is not yet sufficiently investigated to account for several complexities occurring within and between subspecies, thus the potential for evolution towards causing foodborne illness or more serious invasive disease remains [4].

*S. enterica* subspecies I (*S*.), however, is exceptionally scrutinized because of its link to foods commonly consumed by most populations. There are two lineages within subspecies I. Typhoidal and paratyphoidal serotypes are mostly restricted to persistent colonization and infection of people. Approximately 10% of the 20 million infections occurring worldwide on an annual basis cause a life-threatening bacteremia and unusual rash, rather than typical gastrointestinal symptoms, and this set of symptoms is recognized as Typhoid Fever (https://www.who.int/news-room/fact-sheets/detail/typhoid, accessed on 10 May 2022). Typhoidal serotypes most often involve *S. typhi* and *S. paratyphi*, and they are infrequently encountered in the United States, where they occur in association with international travel [5]. In the United States, vaccination against Typhoidal serotypes is recommended and/or required for some international travel.

Non-typhoidal serotypes, exemplified by *S. enteritidis* and *S. typhimurium*, are those frequent and persistent serotypes that are associated with foodborne illness. They are of great epidemiological significance, in part because they persist in food production settings and have the potential to propagate widely and rapidly throughout populations. A few examples include the contamination of nationally distributed ice cream, smoked salmon, chocolate cake, and multistate outbreaks involving cucumbers [6,7,8,9]. Outbreaks with non-typhoidal serotypes of *S. enterica* subspecies 1 can involve thousands of people spread out over a wide geographic area, in part because foods are rapidly disseminated throughout entire countries and even overseas. Attack rates, which is the incidence of clinical illness observed among those exposed, can range widely around 25%, which means hundreds of hospitalizations may result, and clusters can overwhelm hospitals. Another impact from detecting clinical cases is that potentially contaminated products are recalled, which affects the price and availability of food domestically and internationally [10]. Controlling the presence of *S. enterica* subspecies I in products and markets is thus both a food safety and a food security issue.

Approximately 30 of the over 1500 serotypes of *S. enterica* subspecies I are of epidemiological significance, although some variation occurs annually. Other rare serotypes can be encountered as the source of outbreaks but there are often extenuating circumstances involving frank mishandling of food, susceptibility of the affected population, or uncooked products [11,12,13]. Thus, within a single bacterial subspecies, it is evident that selection for only the most fit *Salmonella enterica* subspecies I serotypes capable of causing foodborne illness is stringent. It is also evident that many *S. enterica* serotypes have the potential to cause illness given favorable circumstances; thus, quality control assessments of all foods and food ingredients for people and pets are important.

A few serotypes, primarily *S. enteritidis*, *S. typhimurium*, *S. newport*, *S. infantis*, and *S. javiana*, cause approximately 80% of all of the confirmed cases of salmonellosis each year in the United States [14,15]. *S. enteritidis* became the world’s leading cause of salmonellosis after emerging to prominence in the 1980s, in part because of its abilities to contaminate the contents of eggs produced by otherwise healthy hens, the poultry production environment, and to also maintain the ability to colonize a wide range of food sources, environments, and hosts [16,17]. Finding the genomic determinants of biological capabilities and the environmental persistence of *S. enterica* subspecies I by comparing the genome of the pinnacle pathogen *S. enteritidis* to those from other serotypes is an opportunity for characterizing the “interactome”, which is defined in this review as the correlation between the genotype and phenotype. Determining how *Salmonella* interacts with the environment, people, food animals, plants, and other contributors to foodborne disease will help find new control measures for a pathogen that persistently impacts the safety and security of the food supply.

## 2. Speciation as an Inherent Contributor to *Salmonella* Persistence and Pathobiology

One view of *S. enterica* for bioinformatics purposes is that it is a single genus and species with the taxonomic ID 28901 (TAXID:28901); however, this grouping does not take into account epidemiological considerations related to public health described in the introduction (Search: salmonella enterica-NLM (nih.gov, accessed on 10 May 2022). The degree to which *S. enterica* subspecies I (TAXID:59201) can maintain a consistent genome organization is demonstrated by homopolymer k-mer content of adenine and thymine maintained across serotypes, which occurs even in the presence of inversions, transpositions, lysogenic elements, and generation of single nucleotide polymorphisms (SNPs) between serotypes and variants within serotypes [18]. Whereas *S. enteritidis* can appear to be more homogeneous than other prevalent serotypes causing illness by some measures of genomic content, k-mer analysis confirmed that its genome was not different from that of other subspecies I serotypes [18]; in addition, a mix of less frequently isolated *S. enterica* serotypes had similar k-mer content while other bacterial genera differed substantially. Other studies found confounding information about speciation, which supports that the genome of *S. enterica* has exceptional plasticity [4]. These characteristics of the genome of *S. enterica* suggest that selection pressure to maintain a core genome exists across a multitude of genomic events and time [19,20]. *Salmonella* strains sharing 99% of the genome content appear to vary in readiness to undergo acquisition of new DNA, which could be influenced by the environment, genome structure, and gene expression differences [21,22,23].

When gene conservation rather than placement within the genome of *S. enterica* is the focus, strains that share evolutionary heritage can be identified even when major rearrangements have taken place or different bacteriophage and insertion elements have been acquired [24]. When isolates are obtained within a relatively short time frame, such as often occurs during outbreak investigations, single nucleotide polymorphisms (SNPs) differentiate strains and refine outbreak investigations [25,26]. However, epidemiological evidence is required to connect causation of an outbreak with SNP content. Genes and classes of genes differ in accumulation of SNPs; thus, some housekeeping genes, often required for core metabolic or structural functions, have little room for further evolution. This would mean that SNPs in genes or genomic elements considered core to the biological impact of species carry more weight for differentiation of clades [27,28,29]. Selection pressure placed on pathogens by the host, and the site of colonization within a host, can also limit the amount of genetic variability observed over time. Examples of host selection pressure narrowing genomic variability for *S. enterica* include the relative clonality of egg-contaminating *S. enteritidis* in comparison to other prevalent foodborne serotypes and clonality of the typhoidal *S. enterica* subspecies I of humans, namely *S. typhi* and *S. paratyphi*. In these instances, WGS, or approaches based on knowledge of WGS, is required to detect and characterize SNPs associated with evolutionary trends [30,31,32,33,34].

## 3. Coordinating Schemes for Communicating *S. enterica* Subspecies I Variation

Differentiation of *S. enterica* by the Kaufman–White–LeMinor (KWL) serotyping scheme is perhaps one of the most successful subtyping schemes ever developed, and details are maintained by the Pasteur Institute [35]. Serotyping remains an important classification scheme because epidemiological patterns of disease are often associated with specific serotypes and virulence determinants [36,37]. The scheme predates routine use of whole genome sequencing for analysis of foodborne pathogen by at least 70 years and it is widely accepted as the science-based language for discussing *Salmonella* epidemiology. Serotype designations based on antigenic formulae are useful for epidemiological investigation and ease of language. However, recent molecular studies have confirmed that they are not always necessarily genetically related due to limitation of surface epitopes to convey information about other genomic changes in gene content. In addition, serotypes can accumulate mutations that impair expression of surface antigens thus limiting the use of serological serotyping. In addition, horizontal gene transfer commonly contributes to genetic variation, thus there is a need for methods that recognize genetically related populations. Thus, despite its success, the KWL scheme is not capable of nuanced analysis of evolutionary trends as is whole genome sequencing or other methods based on analysis of DNA. Additional challenges are that reagents are becoming increasingly expensive, and for some antisera, increasingly difficult to acquire. Another problem is that interpreting agglutination reactions have a degree of subjectivity; however, rigorous training and use of high-quality reagents minimizes mistakes in interpretation.

Designated reference laboratories should be supported for maintaining institutional knowledge, training, reagents, and protocols for conducting KWL serotyping. The same reference laboratories could serve as a source of isolates for the purpose of further epidemiological analysis by DNA-based methods with high resolution. This may be an increasingly important function since some agencies are leaning towards Culture Independent Diagnostic Tests (CIDTs). Failure to maintain relevant strain banks could limit access to living organisms in the future for conducting applied research, developing vaccines, and assessing control measures [38]. It is also important that the international language developed through the KWL scheme to discuss *S. enterica* incidence, prevalence, and epidemiology be referenced even as it is refined. Coordinated nomenclature around the world will keep the lines of communication clear because *S. enterica* could again undergo rapid clonal expansion and spread around the world as did serotype *S. enteritidis*. Failure of global cooperation is a threat to public health and the security of the food supply.

### 3.1. Serotype as a Legacy Subtyping Scheme

Of all the subtyping schemes for *Salmonella*, the KWL scheme provides information about the molecules that are physically present on the outer membrane of isolates as they are analyzed in real time. The *Salmonella* serotyping scheme is based on outer membrane epitopes present on the terminal repeating units of lipopolysaccharide and on the flagellar structural proteins, *fliC* and *fljB*. The epitopes are referred to as O- and H-antigens. Some serotypes express two flagellar subunits from genes *fliC* and *fljB*; therefore, the H-antigens are divided into H1 and H2 subsets. Monospecific antisera can be combined to determine unique O:H1:H2 epitope profiles, and examples of immunotype formulas are 1,9,12:g,m: –for most monoflagellated strains of *S. enteritidis* and 1,4,[5],12:i:1,2 for diflagellated *S. typhimurium*.

In the United States and abroad, many small laboratories have access to antisera sets for performing slide agglutination reactions, even if they cannot perform DNA-based analysis. Some DNA-based methods cannot distinguish between closely related variants that differ in their ability to express O- and H-antigens, whereas the KWL scheme can [39]. Variation within a single serotype can also be recognized by the KWL scheme with a range of antisera. For example, *S. enteritidis* is recognized by The Pasteur Institute as having rare variants with factors H1:p, 1H:f, and H1:t; in addition, there is a rare H2:1,7 variant [35]. Thus, it is important that reference laboratories maintain antisera stock and serotyping capabilities because WGS and other DNA-based methods require correlation to historical serotypes. Laboratories performing KWL serotyping should be familiar with a range of DNA-based methods so that difficult strains can be classified by more than one method. It is becoming apparent that naming serotypes should be assessed in a coordinated fashion between countries, because the system of naming each serotype after geographical locations has become unwieldy, misses important details about how *S. enterica* is evolving, and might not be a viable scheme in coming years.

### 3.2. DNA-Based Methods for Coordination with Serotyping Schemes

The legacy multi-locus sequence typing (MLST) hybridization scheme assayed seven loci by PCR amplification, and accessible protocols list primers and gene targets (protocols used for MLST of *Salmonella enterica*—EnteroBase documentation) [40]. The hybridization platform used to evaluate PCR products was automated into commercial platforms because there was no need to obtain sequence; thus, it is still being used for the detection of prevalent *Salmonella* serotypes. Classification is by sequence type (ST), which feeds into evolutionary burst groups (eBG) as data accumulate and is expanded to include WGS and large databases [41]. It is suggested that merging current EnteroBase nomenclature with the KWL scheme is a scaffold for a new system that maintains historical reference, even as it transitions to include greatly expanded databases from WGS. Serotype “*S. typhimurium*” would become “*S. typhimurium* (STXX)” if a rare sequence type, or “*S. typhimurium* (eBGXXX)” if more commonly encountered. Other WGS-based schemes that could impact nomenclature are in progress and international cooperation is suggested to coordinate efforts [36].

### 3.3. Serotyping Is Important for Quality Control as Well as Epidemiological Analysis

Not all uses of serotyping are for epidemiological research or to establish the source of a foodborne outbreak. Many companies, agricultural commodity producers, exporters and importers, and service laboratories want to test feeds for animals, food for consumption, and production environments for purposes of in-house quality control and to assure the safety of their product prior to entering the market [42,43,44]. Enhanced screening regimens that are not regulated are used to provide early warning of emerging problems [45]. Methods for these purposes must have low overhead, minimal need for bioinformatics analysis, and streamlined data management. A commercialized and AOAC certified method used in the EU avoids the need for sequencing by using primers to hybridize target DNA within a microarray. Patterns of spots are read by proprietary imaging equipment and the software assigns the serotype [46,47]. Another method to meet industry needs was developed that uses PCR in a primer-specific two-step process to first locate and then to amplify a single region of the *S. enterica* genome that is linked to over 200 serotypes by generation of a short sequence of about 500 base pairs. The genomic target is the dkgB-linked intergenic sequence ribotype (ISR) region, and the database, protocols, and primers for its use are available through GitHub. Its development was coordinated with DNA hybridization protocols [48]. ISR is a screening method, does not seek certifications, and by design is meant to detect emerging issues without a requirement for reporting. The topic of liability inhibits some companies from performing routine screening for *Salmonella* serotypes more than is required by regulatory agencies such as The Food and Drug Administration and The Food Safety Inspection Service. Finding consensus between government, industry, and consumer groups regarding information that can remain in house versus requiring reporting could encourage more testing in production settings [49].

### 3.4. Surprising Outer Membrane Variation Detected by Serotyping Reagents Is a First Glimpse into Genome and Phenome Interactions

The KWL serotype scheme has some ability to discern unusual phenotypes. When performed as quantitative dilution assays, serotype reagents can be used as a first method to detect strains of *S. enterica* producing a high molecular mass structural variant of the O-antigen region of lipopolysaccharide (HMM LPS) [50,51]. In experimental infections of the egg-laying hen, serotype *S. enteritidis* strains that could produce HMM LPS were better able to contaminate eggs [52]. *S. typhimurium* did not form the structure although it had an otherwise complete O-antigen structure that provided complementary resistance [53]. Other research confirmed the HMM LPS structure and determined some regulatory determinants; thus, HMM LPS that forms a capsule-like structure appears to be a major virulence determinant that contributes to the epidemiology of serotype *S. enteritidis* [54,55]. Serotype *S. enteritidis* produces a surprising variety of O-antigen structures, ranging from no production to producing copious encapsulation [51,56]. Thus, egg-contaminating *S. enterica* likely requires a method for the sensitive regulation of the O-antigen so that the path to the egg completes, while also allowing some embryos within a fertilized egg to survive exposure. We hypothesize, without evidence, that the association between *S. enterica* serotype and poultry is part of a biological cycle where migrating birds adapt to new environments and the demand for reproductive success by acquiring a new serotype impacting the microbiome. LPS is a powerful immuno-modulating molecule and biological toxin, and there is evidence that Aves has evolved different responses than those observed in mammals [57]. The process of molting hens to obtain another cycle of egg production can be a contributor to shedding and spread within flocks [58]. As with O-antigen, the flagellar determinants of serotype also undergo tremendous variation that impacts virulence, environmental spread by swarming and swimming, and microbial communication through some branches of quorum sensing [59,60]. Taken together, these lines of research indicate that the evolution of *S. enterica* to colonize the reproductive tracts of birds and to transmit intergenerationally is a special virulence phenotype that likely requires immense selection pressure to maintain an optimized genome capable of such adaptability. These evolutionary trajectories can be targeted for inhibition and to design strategies for the best use of antibiotics to avoid resistance [61,62].

## 4. The Infection Pathway of *Salmonella enterica* Has Many Evolutionary Detours

The result of the evolutionary path of *S. enteritidis* is contamination of the internal contents of intact shell eggs and a recognized association with consumption of contaminated poultry products and many other food products [63]. However, egg contamination can lose its association with causing foodborne illness, as exemplified by the closely related serotypes *S. gallinarum* and *S. pullorum*. These two serotypes are host-restricted to the bird and are not a cause of human foodborne illness [64]. They are threats to food security because of high transmittance and mortality in chicks and mature birds [65]. In contrast, serotype *S. enteritidis* causes little illness in colonized flocks and also maintains the ability to contaminate other animal products, as well as vegetables and fruits [63,66].

*S. typhimurium* can rival *S. enteritidis* in prevalence at different times and places, and it used to be the most prevalent foodborne serotype. Many molecular biology experiments, analyses of gene function, characterizations of virulence elements, and genomic tooling for the Salmonellae are based on serotype *S. typhimurium* [67,68,69,70,71]. The reference genome defining *Salmonella enterica* subspecies I is from serotype Typhimurium [72]. Currently, the evolution of a monoflagellated variant is of concern, in large part because of its association with multiple antibiotic resistances [73,74,75]. It is of note that *S. enteritidis* is also monoflagellated with rare exceptions. Thus, monoflagellation is a phenotypic trait of concern within the Salmonellae that is perhaps an indicator of evolution towards an infection pathway with potential to impact the safety of food and also animal or human health [76,77,78].

Host restriction is a phenotypic characteristic that occurs within *S. enterica* subspecies I and, as described in the introduction, it is part of the basis for dividing *S. enterica* subspecies I into typhoidal and nontyphoidal groupings, based on how they impact human health. *S. gallinarum* and *S. pullorum*, which cause Fowl Typhoid and White Bacillary Disease, respectively, are restricted to birds. For both human and avian-restricted serotypes, genome degradation occurs and thus the ability to infect a broad range of hosts and grow in a plethora of environments is lost. *S. abortusovis* is host-restricted to sheep and *S. dublin* is host-restricted to being a pathogen of cattle that can cause serious illness in humans. Host restriction is, in general, associated with genome degradation and loss of gene function [5].

In contrast to serotypes that are often associated with foodborne illness, *S.* Kentucky has a very different evolutionary pathway impacting human health. There are two prominent genomic lineages, namely ST198 and ST152, which are, respectively, linked to life-threatening antibiotic resistance and to widespread environmental prevalence in poultry and cattle, but rarely to foodborne illness [79,80,81]. In the United States, ST152 is prevalent, especially in poultry products, and ST198 is primarily associated with travel to Asia, Africa, and other countries where it is endemic [82] (Serotypes Profile of *Salmonella* Isolates from Meat and Poultry Products 1998–2014 (usda.gov, accessed on 10 May 2022). There is genetic evidence that it may have accumulated some mutations shared with other subspecies of *Salmonella* and *Escherichia coli* (*E. coli*) that differentiate it from serotypes commonly associated with foodborne illness [83]. Domestic strains of *S. kentucky* were less invasive than serotype *S. enteritidis* in hens [84]. In summary, *S. kentucky* is an example of a highly successful environmental colonizer within the Salmonellae, even though it is an infrequent cause of foodborne illness.

## 5. Brave New World of Whole Genome Sequencing

Approximately 80 years after the KWL serotyping scheme was first applied and 40 years after serotype *S. enteritidis* emerged as a pathogen of global concern, whole genome sequencing (WGS) has become the gold standard for assessing epidemiological trends of pathogenic organisms [85]. During the decades WGS took to develop, analysis of fragments of DNA produced by restriction enzymes and analyzed by pulsed field gel electrophoresis (PFGE) was used for epidemiological analyses and traceback investigations. The use of PFGE laid the invaluable groundwork of networks across states and countries for tracing the origins of foodborne outbreaks; however, the method could not achieve the resolution required to replace serotyping, to develop different DNA-based approaches to meet different needs, or to analyze genomic polymorphisms occurring at the level of the single nucleotide (PulseNet International: On the Path to Implementing Whole Genome Sequencing for Foodborne Disease Surveillance | International | Participants | PulseNet | CDC). DNA-based methods such as MLST, other platforms using multi-locus gene targeting, ISR, and DNA hybridization, required sequence technology and initial bioinformatics capabilities for their development even as the time and complexity of analysis, database management, and barriers to entry for conducting WGS were reduced. WGS could have perhaps been a major tool for limiting the spread of *S. enteritidis*, and it is imperative that *S. enterica* strains from clinically ill people be sequenced and then the information disseminated rapidly to regulatory and public health agencies. The power of WGS is evidenced by its ability to find previously unrecognized small clusters of human salmonellosis, including geographically dispersed outbreaks [2,9,86,87,88,89].

## 6. Accessing Whole Genome Sequencing and Bioinformatics for Salmonella

The resolution of whole genome sequencing (WGS) to the single base pair enables regulatory agencies, researchers, and public health agencies to conduct unprecedented epidemiological evaluation of the safety of the food supply. For *S. enterica*, large databases have become increasingly available to researchers and submission pathways for sequencing and processing new strains have been streamlined. Within the United States, WGS datasets are routinely deposited by government agencies, domestic and international researchers, and others into the National Center for Biotechnology Information (NCBI) (*Salmonella enterica* (nih.gov, accessed on 10 May 2022). NCBI reviews, curates, and sets standards for accepting the data, which are then made available for public access with some exceptions, and its databases can be accessed by other countries. It has approximately 14,000 draft whole genomes of *S. enterica* in its database, and about 10% are completed (Access date: accessed on 28 April 2022). NCBI also provides some bioinformatics tools, which can be supplemented by other software according to the user’s objectives for bioinformatics analysis.

The coordination of NCBI with EnteroBase has effectively removed many of the barriers to entry for qualified researchers, industry, and public health institutions for access to genomes of *S. enterica*. EnteroBase is a leading international organization for the genotyping of *S. enterica* and other enteric bacteria using multi-locus sequence typing (MLST), and it has a large database of approximately 340,000 *S. enterica* genomes (EnteroBase (warwick.ac.uk, accessed on 10 May 2022). The Food and Drug Administration maintains the GenomeTrkr Network, which is primarily a network of public health and research institutions (GenomeTrakr Network | FDA). Data generated are generally curated and available through the NCBI. One objective is to contribute to the Global Microbial Identifier Network (Forside—Global Microbial Identifier, https://www.globalmicrobialidentifier.org/, accessed on 10 May 2022), which is a consortium envisioning coordination across genome databases, software platforms, data access, and applications to improve food safety. The National Microbiology Laboratory of Canada provides WGS expertise to Canadian researchers and collaborators (National Microbiology Laboratory-Canada.ca). The Wellcome Sanger Institute in the United Kingdom was the first to sequence the human genome, and in regard to *S. enterica*, was the first to sequence the genome of serotype *S. enteritidis*. As its mission expanded towards biomedical research, it produced reference genomes of several pathogens and is especially focused on typhoidal *Salmonella* causing life threatening illness and contributing to antibiotic resistance (*Salmonella*-Wellcome Sanger Institute). It is partnered with the European Molecular Biology Laboratory–European Bioinformatics Institute for the purpose of data mining (EMBL-EBI: European Molecular Biology Laboratory’s European Bioinformatics Institute). EMBL-EBI has services in bioinformatics that are freely available, and resources include portable data and software sharing. The impact of these organizations is the removal of barriers to entry for conducting epidemiological research associated with protecting the health of people and animals.

## 7. Emergent Challenge: Define the Interactome by Correlating Genotype to Phenotype

Identifying that an organism has a gene, or even finding that variants of a gene exist, tells nothing about the function, regulation, or interactions with other genes in the absence of biological assay within in vivo and in vitro systems. There have been advances in approaches for assaying the impact of genomic variability on phenotypic behavior. This type of analysis helps to identify characteristics of the “interactome” of a cell, which is defined by all of the molecular interactions occurring that ultimately produce a discernable phenotype [90,91,92]. Future research could investigate how the interactome of bacteria can be manipulated to improve the immune response of the host to combat colonization. Another application of interactome research for *S. enterica* is the development of novel cancer immunomodulatory therapeutics [93]. The problem of antibiotic resistance will always require experimental approaches to identify alternative methods of eliminating infection and modifying environments to make them safer [94]. In addition, database weighting is needed to accommodate emergent issues that might at first be represented as a rare variant [95]. Researchers suggest that some cells that share a genome with the majority within biofilms can enter different developmental pathways due to epigenetic influences and DNA modifications of either the pathogen or the host [96,97,98].

Under a broader biological umbrella, knowing what differentiates a queen bee from a worker, or a prevalent *Salmonella* serotype from a rare one, cannot be solved by knowledge of DNA sequence alone [99]. Confirmatory experimentation will be the next challenge to link the genotype to phenotype. Here we will discuss three platforms that help to characterize the interactome of *S. enterica*. In addition to these, algorithms that are available as freeware or embedded within some software suites have predictive capabilities with regards to assigning putative gene function, immunogenicity, and regulatory actions. However, all predictive programs require laboratory confirmation within in vitro and in vivo experiments prior to committing resources into product development. An example of the limitation of predictions is that an epitope identified as putatively immunogenic for vaccine development might have deleterious reactions within a host that initiate an allergic or auto-immune reaction [100,101,102].

BioCyc is a database collection and software platform for modeling biochemical pathway utilization by eukaryotes and microbes (BioCyc Pathway/Genome Database Collection) [103,104]. When a gene name is entered into EcoCyc, which is a subset of BioCyc limited to enteric microbe pathways, tables of information are generated about the function, regulation, structure, references, and genomic characteristics including promotor sites. Thus, BioCyc is an efficient way to tap into decades of genetic and biochemical studies that have focused on gene function and interactions.

The PathoSystems Resource Integration Center (PATRIC) is a genomics-centric relational database and bioinformatics resource designed to assist scientists in infectious disease research [105]. The PATRIC platform can also be used in the characterization of *Salmonella enterica*, among other infectious pathogens [106]. It is a good example of a free platform that could be requested as a plugin within subscription software packages.

A third platform is a proprietary phenotype microarray that assesses the ability of bacteria, yeast, and eukaryote cells to metabolize a wide range of compounds and to survive within defined environments (Phenotype MicroArrays for Microbial Cells–Biology) [107]. It differs from EcoCyc and PATRIC in that it is laboratory analysis of function designed in microarray format. To study the metabolome of gram-negative bacteria, the metabolic pathways of E. coli were substantively divided into well format so that the presence of a single metabolite would determine if respiration occurred, and thus confirm that a biochemical pathway was functioning. Absence of function has been used to confirm naturally occurring mutations and changes in global phenotype within *S. enteritidis* [108]. It was expanded to include gradients that would test a range of conditions under which bacteria would respire; thus, it can also be used to study antibiotic and antimicrobial resistances [109,110]. In coordination with WGS it has been used to catalog and describe sets of single nucleotide polymorphisms that contribute to biofilm, fimbria, and ubiquitous utilization of a wide range of metabolites [83].

## 8. Conclusions

Progress in controlling and reducing foodborne illnesses from pathogens such as *S. enterica* subspecies I is critical for protecting public health and assuring the security of the food supply as the world encounters threats from climate change, supply issues, and an increasing population. Whole genome sequencing (WGS) of bacterial pathogens is now a commonly applied epidemiological tool used in outbreaks to identify sources, investigate trends in antibiotic resistances, remove contaminated product, and to limit sickness in people. However, WGS has not yet been fully integrated with molecular biology. Applied research intended to improve vaccines, avoid emergence of antibiotic resistance, and to eliminate pathogens within production systems requires biological confirmation prior to implementation [111]. Therefore, subject areas such as biochemistry, molecular biology, environmental remediation, and pharmaceutical development will remain important, perhaps even more so, as promising information is gleaned from large WGS databases. Thus, biologists grounded in the ability to transfer bioinformatics system data to real world applications will be needed. Algorithms designed to include controls for biological considerations rather than pure mathematical prowess will be a challenge to develop because experts in several specialties will need to input the parameters. The potential for alternative modes of gene expression from one genome poses a limit to how WGS can be applied for solving biological issues.

## Data Availability

Not applicable.
